# Synthesis, Characterization and Protonation Behavior of Quinoxaline-Fused Porphycenes

**DOI:** 10.3390/molecules22060908

**Published:** 2017-05-31

**Authors:** Daiki Kuzuhara, Mika Sakaguchi, Wataru Furukawa, Takuya Okabe, Naoki Aratani, Hiroko Yamada

**Affiliations:** Graduate School of Materials Science, Nara Institute of Science and Technology, 8916-5 Takayama-cho, Ikoma 630-0192, Japan; uzusio.ampm@gmail.com (M.S.); furukawa.wataru.fq3@ms.naist.jp (W.F.); okabe.takuya.oj3@ms.naist.jp (T.O.); aratani@ms.naist.jp (N.A.)

**Keywords:** porphycene, π-expansion, NIR absorption and fluorescence, protonation

## Abstract

9,10-Quinoxaline-fused porphycenes **1a-H_2_** and **1b-H_2_** were synthesized by intramolecular McMurry coupling. As a result of the annulation of the quinoxaline moiety on the porphycene skeleton, **1a-H_2_** and **1b-H_2_** display absorption and fluorescence in the near infra-red (NIR) region. Additionally, the quinoxaline moieties of **1a-H_2_** and **1b-H_2_** act as electron-withdrawing groups, introducing lower reduction potentials than for pristine porphycene. The protonation occurred at the nitrogen atoms in the cavity of freebase porphycenes and at the quinoxaline moieties for their nickel complexes to give diprotonic species.

## 1. Introduction

**Figure 1:**
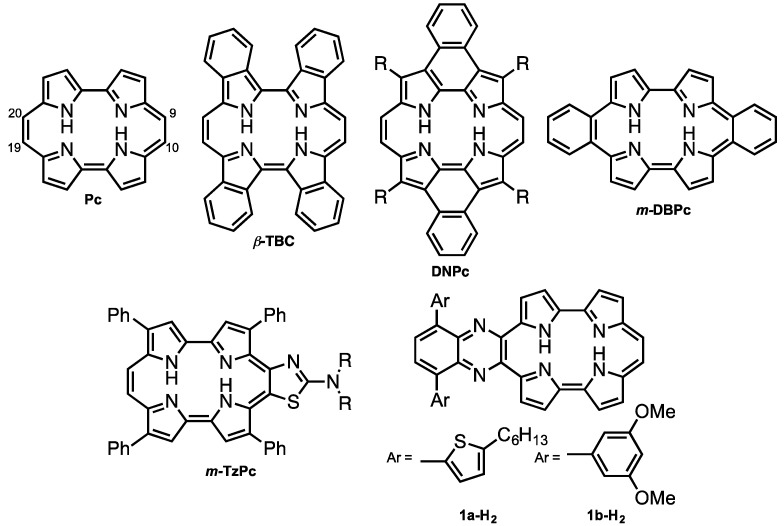
Structures of porphycene (**Pc**) and π-expanded porphycenes.

Porphycene (**Pc** < Figure 1) is the first reported constitutional isomer of porphyrin [1]. It has a rectangular-shaped structure with two bipyrrole units connected by two ethylene bridges. This structural change leads to a lower LUMO level and narrower HOMO-LUMO energy difference compared with those of porphyrin [2]. In addition, porphycene derivatives and their metal complexes are capable of generating singlet oxygen efficiently [3,4]. As a result of these electronic and optical properties, porphycene derivatives are applied as photosensitizers for photodynamic therapy [5,6] and non-linear optical materials [7,8,9,10,11]. However, there have been only a few reports on porphycenes having near infra-red (NIR) absorption, although NIR absorbing materials can be utilized in organic solar cells, biomarkers and photodynamic reagents. Benzoannulation has great potential to expand the π-conjugations and achieve NIR absorptions [12]. We have previously reported on tetrabenzo-porphycene (β-TBPc, Figure 1) [13] and tetranaphthoporphycene (β-TNPc) [14], which demonstrated red-shifted absorption from pristine **Pc** with an increasing number of benzene rings. Organic solar cells (OSC) based on β-TBPc as a p-type material and PC_61_BM as an n-type material exhibited 1.54% power-conversion efficiency [15,16,17]. Sessler and Panda independently reported dinaphthoporphycenes (DNPcs), in which absorptions reached around 800 nm [8,18]. Furthermore, the π-conjugation of porphycenes can be expanded by benzoannulation at *meso*-positions (9, 10, 19 and 20-positions, Figure 1). *meso*-Monobenzobenzoporphycene (*m*-MBPc) showed an absorption peak at 762 nm [19]. Recently, Hayashi reported *meso*-dibenzoporphycene (***m*-DBPc**), displaying an extraordinarily red-shifted absorption peak at 1047 nm [20], indicating that benzoannulation at the *meso*-positions earns the largest perturbations to π-conjugation of porphycenes. In addition, Nonell et al. reported *meso*-thiazole-fused porphycenes (*m*-TzPc), which also showed NIR absorption and fluorescence [21]. In this context we have designed *meso*-quinoxaline-fused porphycene and its nickel complex to attain NIR absorption and fluorescence. Quinoxaline can be employed not only as an electron-withdrawing group to stabilize LUMO energy but also for the expansion of the π-conjugations. Moreover, since modification of the quinoxaline moiety could impact electronic properties and increase solubility, 5-hexylthienyl (**a**) and 3,5-dimethoxyphenyl (**b**) groups are introduced on the quinoxaline unit. Investigation of optical and electrochemical properties, X-ray diffraction analysis, and protonation characteristics are also described.

## 2. Results and Discussion

### 2.1. Synthesis and Characterization of Quinoxaline-Fused Porphycenes and Their Nickel Complexes

**Scheme 1:**
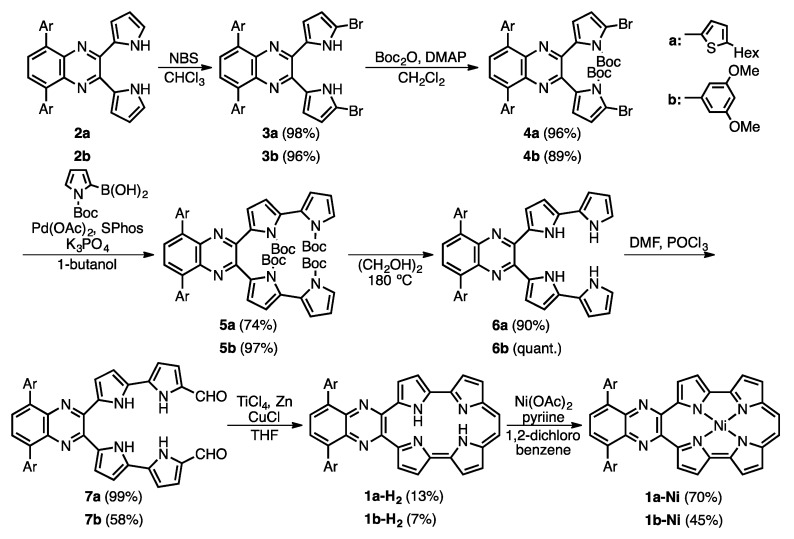
Synthesis of **1a-H_2_**, **1b-H_2_**, **1a-Ni** and **1b-Ni**.

Scheme 1 shows the synthetic scheme of **1a-H_2_**, **1b-H_2_** and their nickel complexes, **1a-Ni** and **1b-Ni**. The starting materials, **2a** and **2b**, were synthesized according to established procedures [22,23]. The bromination of **2a** and **2b** with *N*-bromosuccinimide (NBS) gave **3a** and **3b** in 98 and 96% yield, respectively, and then protection at the pyrrolic NH groups with *tert*-butoxycarbonyl (Boc) groups gave **4a** and **4b** in 96% and 89%, respectively. To synthesize the 2,2′-bipyrrole units, Suzuki-Miyaura coupling conditions were optimized. After screening coupling conditions, we found that **5a** and **5b** were obtained with Boc-protected pyrrole-2-boronic acid, Pd(OAc)_2_, 2-dicyclohexylphosphino-2′,6′-dimethoxybiphenyl (SPhos) and K_3_PO_4_ in 1-butanol in 74% and 97%, respectively. The similar coupling conditions were reported by Buchwald [24]. After deprotection of the Boc groups of **5a** and **5b** under heating conditions in ethylene glycol, formylation by Vilsmeier-Haack reaction of **6a** and **6b** gave **7a** and **7b**, in 99% and 58%, respectively. Finally, an intramolecular McMurry coupling of **7a** and **7b** afforded the quinoxaline-fused porphycenes, **1a-H_2_** and **1b-H_2_**, in 13% and 7%, respectively. The corresponding nickel complexes, **1a-Ni** and **1b-Ni**, were synthesized with Ni(OAc)_2_ in 1,2-dichlorobenzene in the presence of pyridine in 70% and 45%, respectively. These compounds were characterized by ^1^H-, ^13^C-NMR and mass spectroscopies and X-ray crystallography. The high-resolution mass spectroscopy detected parent ion peaks of **1a-H_2_** and **1b-H_2_** at *m*/*z* = 745.3147 (calcd. for C_46_H_42_N_6_S_2_: 745.3169 [M]^+^) and 685.4412 (calcd. for C_40_H_35_O_4_N_5_Na: 685.4426 [M + Na]^+^), respectively.

The single crystal X-ray structures of **2a** and **2b** are shown in Figure S1 in Supplementary Materials. The NH groups of pyrroles face nitrogen atoms in the quinoxaline moiety in the formation of the hydrogen bondings. The thiophenes and quinoxaline of **2a** consist of relatively planar structures with twisted angles 12.35° and 10.38° facilitating nitrogen-sulfur interactions. On the other hand, **2b** shows larger twisted angles (40.82° and 47.07°) between dimethoxybenzenes and quinoxaline.

**Figure 2:**
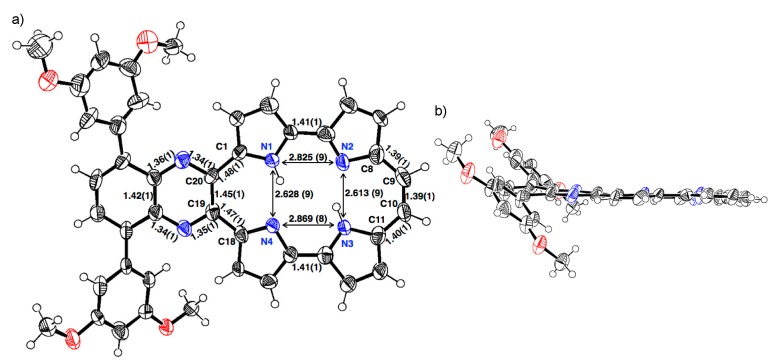
Crystal structure of **1b-H_2_**. (**a**) Top view and selected bond lengths; (**b**) side view. Thermal ellipsoids represent for 50% probability.

The single crystal of **1b-H_2_** was obtained from a mixture of THF/methanol/trifluoroacetic acid (TFA) (Figure 2). The porphycene core forms a highly planar structure in which a mean-plane deviation of 24 porphycene atoms is 0.046 Å. The porphycene and quinoxaline units are slightly tilted and the dihedral angle of each plane is 8.07°. The 3,5-dimethoxybenzene on quinoxaline moiety makes the angles of 45.10° and 45.38°, which are consistent with those of **2b**. The distances of N1–N4 and N2–N3 are 2.628(9) and 2.613(9) Å, respectively, which values are comparable with that of **Pc** (2.63 Å) [1] and longer than those of *m*-MBPc (2.578 and 2.589 Å) and *m*-DBPc (2.54 Å) [20]. The distances of C8–C9 (1.39(1) Å) and C10–C11 (1.40(1) Å) are consistent with those of **Pc** (<1.40 Å) [1]. In contrast, the bond lengths of the opposite side, C1–C20 and C18–C19 exhibit slightly longer distances (1.48(1) and 1.47(1) Å).

**Figure 3:**
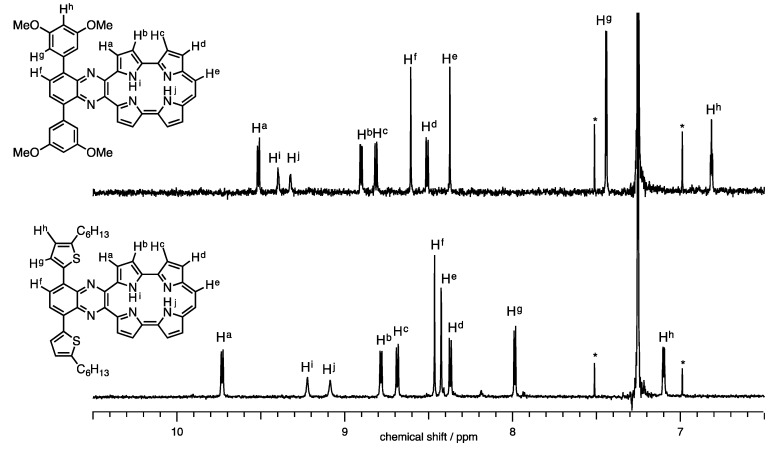
^1^H-NMR spectra of **1a-H_2_** and **1b-H_2_** in CDCl_3_.

**Figure 4:**
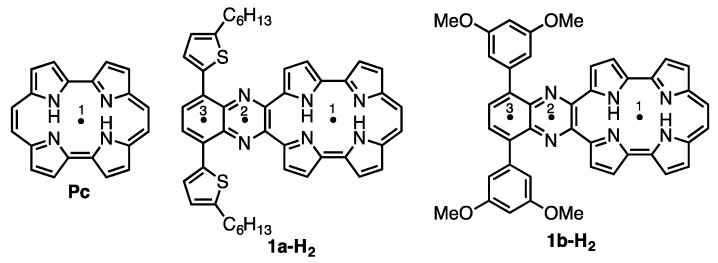
NICS(0) values of **Pc**, **1a-H_2_** and **1b-H_2_**.

The ^1^H-NMR spectra of **1a-H_2_** and **1b-H_2_** in CDCl_3_ are shown in Figure 3. Full characterizations were carried out with H-H COSY and NOE techniques (Figures S2 and S3). The NH proton peaks were observed at 9.05 and 9.17 ppm for **1a-H_2_** and 9.32 and 9.40 ppm for **1b-H_2_**. Generally, NH chemical shifts of porphycenes display in the range of 1 to 7 ppm depending on the substituents with change in cavity sizes and the effect of the hydrogen bond [20,25]. The NH chemical shifts of **1a-H_2_** and **1b-H_2_** remarkably downfield-shifted from the pristine **Pc**, while the distances of **1b-H_2_** (2.628(9) and 2.613(9) Å), which were obtained by X-ray diffraction, are comparable to **Pc** (2.63 Å). Notably, the chemical shift of the inner NH protons of porphyrinoids is also affected with macrocyclic diatropic ring currents. To interpret the aromaticity of **1a-H_2_** and **1b-H_2_**, the nucleus-independent chemical shift (NICS(0)) values were calculated at the B3LYP/6-31G* level (Figure 4) [26]. The NICS(0) values of the midpoint made by the four nitrogen atoms are –8.71 ppm for **1a-H_2_**, −8.57 ppm for **1b-H_2_** and −15.62 ppm for **Pc**. The result of the attachments of quinoxaline to porphycene induces the decrease in the aromaticities of **1a-H_2_** and **1b-H_2_**, reflecting downfield shifts of the NH protons compared with the pristine **Pc**. In addition, the peaks of ethylenic protons of **1a-H_2_** and **1b-H_2_** were observed at 8.42 and 8.37 ppm, respectively. These peaks exhibit an upfield shift compared with the pristine **Pc** (δ = 9.67 ppm) [1]. This result also coincides with the NICS values.

**Figure 5:**
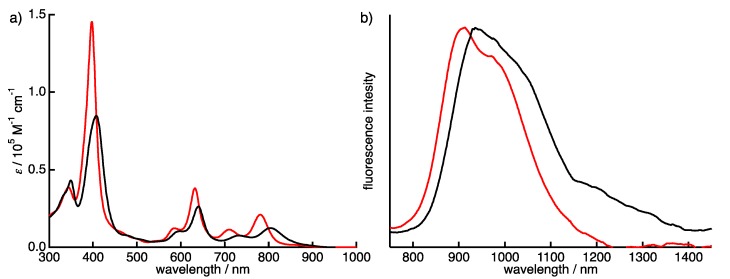
(**a**) Absorption and (**b**) fluorescence spectra of **1a-H_2_** (black) and **1b-H_2_** (red) in CH_2_Cl_2_. The fluorescence was obtained by excitation at 411 nm for **1a-H_2_** and 377 nm for **1b-H_2_**.

The absorption and fluorescence spectra of **1a-H_2_** and **1b-H_2_** were measured in CH_2_Cl_2_ (Figure 5). Typically, porphycene exhibits strong Soret bands around 370 nm and three Q-bands peak-tops at 550 to 660 nm. The **1a-H_2_** and **1b-H_2_** show significantly red-shifted absorption compared with **Pc** due to the expansion of the π-conjugation attached with quinoxaline moieties on the porphycene core. The Soret bands of **1a-H_2_** and **1b-H_2_** are observed at 407 and 397 nm, respectively, associated with four Q-bands peak-tops. The longest absorption peaks are detected at 814 nm for **1a-H_2_** and 781 nm for **1b-H_2_**. Furthermore, the absorption of **1a-H_2_** shows red-shift compared with that of **1b-H_2_** since the coplanarity between thiophene and quinoxaline moiety is higher than that of the 3,5-dimethoxy-benzene of **1b-H_2_**. This indicates that the optical property could be customized by the substitutions on the quinoxaline moiety. The compounds **1a-H_2_** and **1b-H_2_** show fluorescence at the NIR regions. The fluorescence peak tops of **1a-H_2_** and **1b-H_2_** are observed at 935 and 911 nm with quantum yields at 0.16% and 0.20%, as determined by the indocyanine green standards [27].

**Figure 6:**
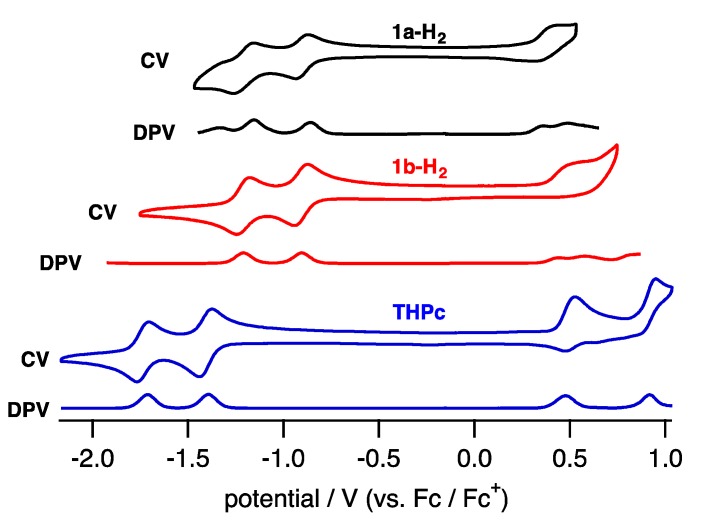
CV and DPV of **1a-H_2_** (black), **1b-H_2_** (red) and **THPc** (blue) in CH_2_Cl_2_.

To elucidate the electronic properties, cyclic voltammetry (CV) and differential pulse voltammetry (DPV) in CH_2_Cl_2_ containing tetrabutylammonium hexafluorophosphate (TBAPF_6_) as an electrolyte were performed (Figure 6). The compounds **1a-H_2_** and **1b-H_2_** exhibited two reversible reduction waves and one quasi-reversible oxidation wave.

**Figure 7:**
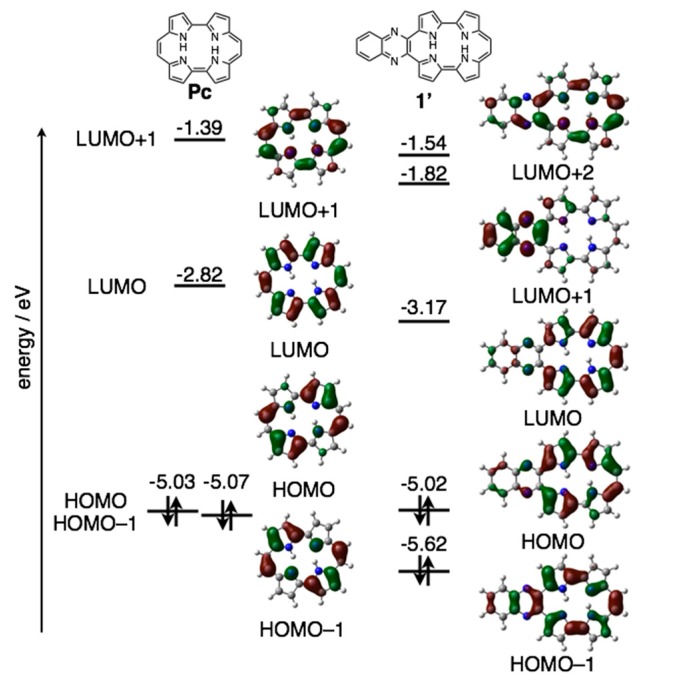
Kohn-Sham molecular orbitals and energy diagrams of **Pc** and **1′**.

The reduction waves are observed at –0.85 and −1.16 V (vs. ferrocene/ferrocenium cation) for **1a-H_2_** and −0.93 and −1.23 V for **1b-H_2_**, while oxidation waves are observed at 0.36 V for **1b-H_2_** and 0.44 V for **1b-H_2_**. In particular, reduction potentials are positively shifted compared with 2,7,12,17-tetrahexylporphycene (−1.42 and −1.74 V) as a reference compound at the same conditions, affecting the attached quinoxaline as an electron-withdrawing group. To further understand the electronic features, DFT calculations were carried out at the B3LYP/6-31G* level using the Gaussian 09 program (Figure 7) [28]. Geometry optimization calculations were carried out using the structure of **1′** with the substituents on quinoxaline moiety removed for simplicity. The energy level of **1′** LUMO is largely stabilized from porphycene (ΔeV = −0.35 eV), while that of HOMO is comparable (ΔeV = −0.01 eV). These values are consistent with the CV results.

### 2.2. Protonation of Quinoxaline-Fused Porphycenes and Their Nickel Complexes

**Figure 8:**
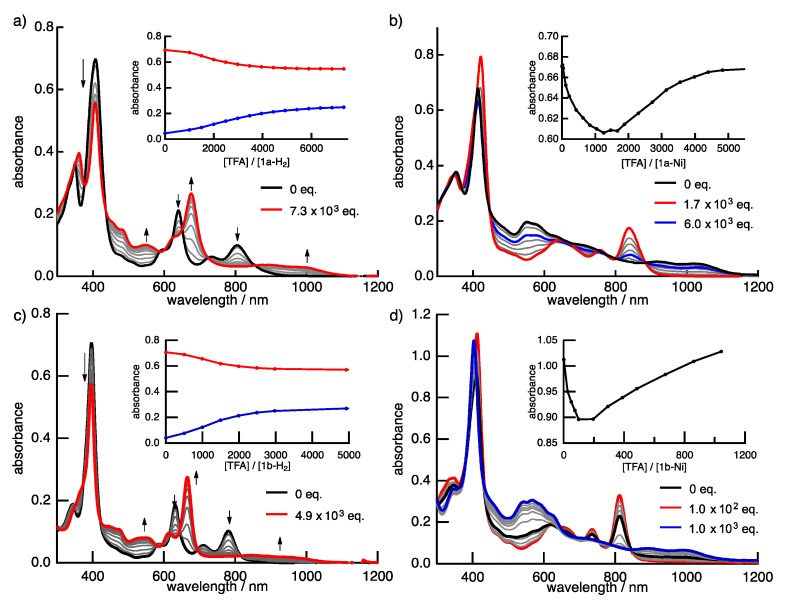
The TFA titration of the absorption spectra of (**a**) **1a-H_2_**, (**b**) **1a-Ni**, (**c**) **1b-H_2_** and (**d**) **1b-Ni** in CH_2_Cl_2_. Inset: absorbance changes at (**a**) 405 (red) and 667 nm (blue); (**b**) at 411 nm; (**c**) 397 (red) and 665 nm (blue), and (**d**) 407 nm.

**Scheme 2:**
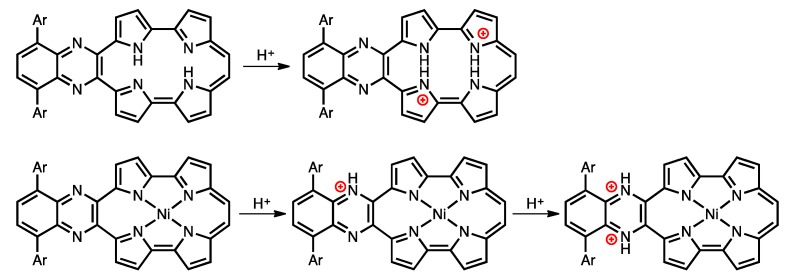
Protonation of free-base and nickel complex of porphycenes.

Protonation is an important phenomenon in porphyrin and related compounds because of its influence on optical and electronic properties [29]. Fukuzumi reported the protonation of distorted octaphenylphthalocyanine and its zinc complex [30]. Interestingly, they reported different phenomena: the freebase octaphenylphthalocyanine was protonated on nitrogen atoms on the inside of the cavity, while the zinc complex was protonated on the *meso*-nitrogen atoms, depending on the basicity. Furthermore, the protonation behavior of porphycene is known to be different from that of porphyrin. The protonation of porphyrin proceeds step-by-step, but the protonation of porphycene gives a porphycene dication immediately. This is because the monoprotonated porphycene is unstable due to the steric repulsion between the neighboring hydrogen atoms and the loss of the hydrogen bonding between NH and N [31,32]. Encouraged by these results, the protonation of **1a-H_2_**, **1b-H_2_** and their nickel complexes was investigated, since the compounds have two different imine-type nitrogen atoms at the cavity and quinoxaline parts. Firstly, protonation was performed for the reference compounds, **THPc** and its nickel complex (**THPc-Ni**). **THPc** showed single absorption change according to the increase in the amount of TFA, while **THPc-Ni** exhibited no spectral changes after the addition of 2120 eq. of TFA because of the absence of protonation sites. (Figures S4 and S5) The changes in the **1a-H_2_** and **1a-Ni** absorption spectra by the TFA titrations are shown in Figure 8. Upon addition of TFA to a solution of **1a-H_2_** in CH_2_Cl_2_, the Soret band at 397 nm decreases simultaneously with the observed Q-like bands at 656 nm and broadened peak over 1000 nm. The isosbestic points are observed at 437, 649, 721 and 853 nm. After the addition of 7300 equivalents of TFA, there are no further changes. In contrast, when TFA is added to a solution of **1a-Ni**, a two–step spectral change is observed. The first spectral change is observed until the addition of 1700 eq. TFA. The Soret band peak at 422 nm decreases and shifts to 419 nm associated with the generation of the broad band around 1100 nm. Subsequently, the second spectral change occurs with the increase in the amount of TFA (1700 eq. to 6000 eq.). The peak at 419 nm is shifted to 412 nm, which slightly increases intensity. The compounds **1b-H_2_** and **1b-Ni** exhibited similar results (Figure 8). These findings suggest that the protonation of the freebase **1a-H_2_** and **1b-H_2_** provide diprotonated porphycene species because these trends correspond to general freebase porphycenes [31,32], while the protonation of nickel complexes give diprotonated quinoxaline species as stepwise reactions (Scheme 2).

## 3. Materials and Methods

### 3.1. General Information

Commercially available reagents and solvents for syntheses were of reagent grade and used without further purification. TLC and gravity column chromatography were performed on Art. 5554 (Merck KGaA, Darmstadt, Germany) plates and silica gel 60N (Kanto Chemical, Chuo, Japan), respectively. For spectral measurements, spectral-grade dichloromethane was purchased from Nacalai Tesque. ^1^H- and ^13^C-NMR spectra were recorded with a JNM-ECX 400 spectrometer JEOL Akishima, Japan) at ambient temperature using tetramethylsilane (TMS) as an internal standard. Electrospray ionization (ESI) mass spectra were recorded on a JEOL JMS-MS T100LC spectrometer. Matrix assisted laser desorption/ionization (MALDI)-TOF-MS spectra were recorded on a JEOL SpiralTOF JMS-S3000 spectrometer (JEOL Akishima, Japan). FAB mass spectra were measured on a JEOL JMS-700 MStation spectrometer. UV-vis spectra were measured using a V-670 UV/VIS/NIR Spectro-photometer (JASCO, Hachioji, Japan). The fluorescence spectra were measured with a HORIBA (Kyoto, Japan) Jobin Yvon Spectrofluorometer Fluorolog-3 (Model: FL3-11-NIR).

### 3.2. X-ray Crystallography

X-ray crystallographic data for **2a** and **2b** were recorded at 90 K on an APEX II X-ray diffractometer (Bruker, Billerica, MA, USA) equipped with a large area CCD detector and graphite monochromated Mo-Kα radiation and the data for **1b-H_2_** and **1a-H_2_** were recorded at 103 K on a R-AXIS RAPID/S instrument (Rigaku, Akishima, Japan) using Mo-Kα radiation. The structure was solved by direct methods with SHELXS-97 [33] and refined by full-matrix least-squares techniques against *F*^2^ (SHELXL-97) [33]. The intensities were corrected for Lorentz and polarization effects. The non-hydrogen atoms were refined anisotropically. Hydrogen atoms were refined using the riding model. All calculations were performed using Crystal Structure 4.0 or Yadokari-XG 2011. Crystallographic data for **1b-H_2_**: C_42_H_32_N_6_O_4_, *Mw* = 684.74, orthorhombic, space group *Pna*2_1_ (#33), *a* = 28.452, *b* = 30.414, *c* = 3.827 Å, *V* = 3311.3 Å^3^, *T* = 103(2) K, *Z* = 4, reflections measured 39,848, 5127 unique. The final *R*_1_ was 0.0880 (*I* > 2σ(*I*)), and the final *wR* on *F*^2^ was 0.2299 (all data), GOF = 1.019. CCDC No. 1543213 contains the supplementary crystallographic data for this paper. These data can be obtained free of charge via http://www.ccdc.cam.ac.uk/conts/retrieving.html (or from the CCDC, 12 Union Road, Cambridge CB2 1EZ, UK; Fax: +44 1223 336033; E-mail: deposit@ccdc.cam.ac.uk).

### 3.3. Cyclic Voltammetry and Differential Pulse Voltammetry Measurements

CV and DPV measurements were examined in a solution of 0.1 M TBAPF_6_ in dry CH_2_Cl_2_ with a scan rate of 100 mV s^−1^ at room temperature in an argon-flowed cell. A glassy carbon electrode and platinum wire were used as a working and counter electrode, respectively. An Ag/AgNO_3_ electrode was used as a reference electrode, which was externally calibrated with the half-wave potential of ferrocene/ferrocenium redox couple.

### 3.4. DFT Calculations

Geometries were fully optimized using the density functional theory (DFT). The functional and basis set used in the DFT calculations were the Becke’s three-parameter hybrid functional combined with the Lee–Yang–Parr correlation functional (B3LYP) and the 6-31G(d) basis set, respectively. Equilibrium geometries were verified via frequency calculations, where no imaginary frequency was found. The excitation energies and oscillator strengths were computed with time-dependent density functional theory (TD-DFT) based on the B3LYP/6-31G(d) optimized geometries. All the calculations were carried out using the Gaussian 09 program suite [28].

### 3.5. Synthesis of 5,8-Bis(5-hexylthiophen-2-yl)-2,3-di(1H-pyrrol-2-yl)quinoxaline *(**2a**)*

5,8-Dibromo-2,3-di(1*H*-2-pyrrolyl)quinoxaline [34] (300 mg, 0.72 mmol), tributyl(5-hexylthiophen-2-yl)stannane (1.27 g, 2.8 mmol) and Pd(PPh_3_)_4_ (80 mg, 0.07 mmol) were dissolved in degassed toluene (15 mL) under an argon atmosphere. The resulting solution was refluxed for 20 h. After cooling to room temperature, the solvent was removed under a reduced pressure. The crude product was purified by silica gel column chromatography (CH_2_Cl_2_/hexanes = 1/1) to give **2a** as an orange solid. Yield: 80% (340 mg, 0.57 mmol). ^1^H-NMR (DMSO-*d*_6_, 400 MHz): δ 10.94 (2H, brs, NH), 8.04 (2H, s), 7.77 (2H, d, *J* = 3.7 Hz), 7.08 (2H, m), 6.89 (2H, d, *J* = 3.7 Hz), 6.39 (2H, m), 6.19 (2H, m), 2.85 (4H, t, *J* = 7.8 Hz), 1.66 (4H, quin., *J* = 7.8 Hz), 1.38–1.26 (12H, m), 0.84 (6H, t, *J* = 7.8 Hz); ^13^C-NMR (CDCl_3_, 100 MHz): δ 148.78, 141.39, 136.15, 135.54, 130.02, 129.70, 125.64, 125.58, 123.91, 121.72, 113.44, 110.08, 32.80, 31.77, 30.31, 29.02, 22.73, 14.25; HRMS (ESI): *m*/*z* 615.2592 (calcd. for C_36_H_40_N_4_NaS_2_ [M + Na]^+^, 615.2593).

### 3.6. Synthesis of 2,3-Bis(5-bromo-1H-pyrrol-2-yl)-5,8-bis(5-hexylthiophen-2-yl)quinoxaline *(**3a**)*

NBS (180 mg, 1.0 mmol) was added to a solution of **2a** (300 mg, 0.50 mmol) in CHCl_3_ (30 mL) under an argon atmosphere. The solution was stirred for 3 h at room temperature under dark condition. The reaction was quenched with water and extracted with CHCl_3_. The combined organic layer was washed with brine and dried over Na_2_SO_4_. After removal of the solvent under a reduced pressure, the crude product was purified by silica gel column chromatography (CH_2_Cl_2_). Recrystallization from CH_2_Cl_2_/methanol gave **3a** as a yellow solid. Yield: 98% (350 mg, 0.47 mmol). ^1^H-NMR (CDCl_3_, 400 MHz): δ = 10.04 (2H, brs, NH), 7.88 (2H, s), 7.46 (2H, d, *J* = 3.7 Hz), 7.18 (2H, dd, *J* = 4.1 and 2.7 Hz), 6.85 (2H, d, *J* = 3.7 Hz), 6.24 (2H, dd, *J* = 4.1 and 2.7 Hz), 2.93 (4H, t, *J* = 7.8 Hz), 1.84 (4H, quin., *J* = 7.8 Hz), 1.49–1.31 (12H, m), 0.90 (6H, t, *J* = 7.8 Hz); ^13^C-NMR (CDCl_3_, 100 MHz): δ 149.26, 139.69, 135.49, 135.20, 130.79, 129.72, 125.49, 125.39, 123.80, 114.47, 112.10, 103.63, 31.88, 31.75, 30.44, 29.19, 22.76, 14.24; HRMS (ESI): *m*/*z* 771.0802 (calcd. for C_36_H_38_Br_2_N_4_NaS_2_ [M + Na]^+^, 771.0807).

### 3.7. Synthesis of Di-tert-butyl 5,5′-(5,8-bis(5-hexylthiophen-2-yl)quinoxaline-2,3-diyl) bis(2-bromo-1H-pyrrole-1-carboxylate) *(**4a**)*

A solution of **3a** (150 mg, 0.22 mmol), DMAP (2 mg, 0.02 mmol) and Boc_2_O (105 mg, 0.48 mmol) in CH_2_Cl_2_ (10 mL) was stirred for 5 h at room temperature under an argon atmosphere. After removal of solvent under a reduced pressure, the crude product was purified by silica gel column chromatography (CH_2_Cl_2_/hexanes = 1/1) to give **4a** as an orange solid. Yield: 96% (184 mg, 0.19 mmol). ^1^H-NMR (CDCl_3_, 400 MHz): δ 7.97 (2H, s), 7.67 (2H, d, *J* = 3.7 Hz), 6.81 (2H, d, *J* = 3.7 Hz), 6.32 (2H, d, *J* = 3.7 Hz), 6.26 (2H, d, *J* = 3.7 Hz), 2.84 (4H, t, *J* = 7.3 Hz), 1.72 (4H, quin., *J* = 7.8 Hz), 1.42–1.30 (12H, m), 1.16 (18H, s), 0.89 (6H, t, *J* = 7.8 Hz); ^13^C-NMR (CDCl_3_, 100 MHz): δ 148.67, 147.75, 144.70, 136.90, 136.40, 133.25, 131.08, 127.48, 127.22, 124.47, 116.30, 115.42, 104.33, 85.36, 31.73, 31.65, 30.30, 29.01, 27.49, 22.73, 14.25; HRMS (FAB): *m*/*z* 948.1953 (calcd. for C_46_H_54_O_4_Br_2_N_4_S_2_ [M]^+^, 948.1948).

### 3.8. Synthesis of Tetra-tert-butyl 5,5″-(5,8-bis(5-hexylthiophen-2-yl)quinoxaline-2,3-diyl) bis(1H,1′H-[2,2′-bipyrrole]-1,1′-dicarboxylate) *(**5a**)*

A degassed solution of (1-(*tert*-butoxycarbonyl)-1*H*-pyrrol-2-yl)boronic acid [26] (19 mg, 0.09 mmol), **4a** (30 mg, 0.031 mmol), Pd(OAc)_2_ (0.3 mg, 0.001 mmol), SPhos (0.8 mg, 0.002 mmol), K_3_PO_4_ (25.5 mg, 0.12 mmol) in water (0.8 mL) and in 1-butanol (2.0 mL) was heated at 80 °C for 20 h under an argon atmosphere. After cooling to room temperature, the reaction mixture was extracted with CHCl_3_. The combined organic layer was washed with brine and dried over Na_2_SO_4_. After removal of the solvent under a reduced pressure, the crude product was purified by silica gel column chromatography (CH_2_Cl_2_/hexanes = 1/1) to give **5a** as an orange solid. Yield: 74% (24 mg, 0.02 mmol). ^1^H-NMR (CDCl_3_, 400 MHz): δ 7.95 (2H, s), 7.75 (2H, d, *J* = 3.7 Hz), 7.43 (2H, m), 6.77 (2H, d, *J* = 3.2 Hz), 6.45 (2H, s), 6.20–6.25 (6H, m), 2.82 (4H, t, *J* = 7.8 Hz), 1.70 (4H, m), 1.42–1.29 (30H, m), 1.06 (18H, s), 0.89 (6H, t, *J* = 6.9 Hz); ^13^C-NMR (CDCl_3_, 100 MHz) (typical signals because some signals were overlapped]: δ 178.7, 144.6, 142.3, 137.8, 137.7 137.5, 132.3, 129.7, 128.2, 126.0, 121.7, 110.9, 45.7, 21.1; HRMS (ESI): *m*/*z* 1145.5220 (calcd. for C_64_H_78_O_8_N_6_NaS_2_ [M + Na]^+^, 1145.52212)

### 3.9. Synthesis of 2,3-Di(1H,1′H-[2,2′-bipyrrol]-5-yl)-5,8-bis(5-hexylthiophen-2-yl)quinoxaline *(**6a**)*

A solution of **5a** (30 mg, 0.027 mmol) in ethylene glycol (10 mL) was degassed for 1 h under a reduced pressure and then purged with an argon gas and heated at 170 °C for 2 h. After cooling to room temperature, the reaction mixture was diluted with water and extracted with CHCl_3_. The combined organic layer was washed with water and brine, then dried over Na_2_SO_4_. After removal of the solvent under a reduced pressure, the crude product was recrystallized from CH_2_Cl_2_/methanol to give **6a** as a red solid. Yield: 92% (18 mg, 0.025 mmol). ^1^H-NMR (DMSO-*d*_6_, 400 MHz): δ 11.34 (2H, brs), 10.88 (2H, brs), 8.11 (2H, s), 7.81 (2H, d, *J* = 3.7 Hz), 6.95 (2H, d, *J* = 3.7 Hz), 6.87 (2H, brs), 6.77 (2H, m), 6.49 (4H, m), 6.13 (2H, dd, *J* = 5.7, 2.5 Hz), 2.89 (4H, t, *J* = 7.8 Hz), 1.71 (6H, t, *J* = 7.6 Hz), 1.34 (12H, m), 0.87 (6H, t, *J* = 7.1 Hz); ^13^C-NMR (CDCl_3_, 100 MHz): δ 148.86, 142.81, 135.67, 135.43, 130.67, 129.52, 128.40, 126.69, 125.70, 125.16, 124.77, 119.40, 114.67, 109.23, 105.61, 104.95, 31.95, 31.49, 30.11, 28.92, 22.64, 14.50; HRMS (ESI): *m*/*z* 745.3123 (calcd. for C_44_H_46_N_6_NaS_2_ [M + Na]^+^, 745.31252).

### 3.10. Synthesis of 5′,5‴-(5,8-Bis(5-hexylthiophen-2-yl)quinoxaline-2,3-diyl)-bis((1H,1′H-[2,2′-bipyrrole]-5-carbaldehyde)) *(**7a**)*

POCl_3_ (0.04 mL, 0.46 mmol) was slowly added to DMF (0.3 mL, 3.9 mmol) at 0 °C under an argon atmosphere to prepare the Vilsmeier’s reagent. A solution of **6a** (17 mg, 0.02 mmol) in DMF (1.0 mL) was added to the Vilsmeier’s reagent at 0 °C. The mixture was stirred for 1 h at 0 °C. The concentrated aqueous acetyl acetate was added to the reaction mixture, followed by stirring for 4 h at room temperature. The reaction mixture was extracted with CHCl_3_. The reaction mixture was extracted with CHCl_3_ and the combined organic layer was washed with water and brine, and then dried over Na_2_SO_4_. The solvent was removed under a reduced pressure. The crude product was recrystallization from CH_2_Cl_2_/methanol to give **7a** as a red solid. Yield: 99% yield (15 mg, 0.02 mmol). ^1^H-NMR (DMSO-*d*_6_, 400 MHz): δ 12.74 (2H, s), 11.52 (2H, s), 9.44 (2H, s), 8.18 (2H, s), 7.85 (2H, d, *J* = 3.8 Hz), 7.09 (2H, dd, *J* = 3.8, 2.2 Hz) 6.90 (2H, t, *J* = 3.0 Hz), 6.75 (2H, dd, *J* = 3.8, 2.2 Hz), 6.70 (2H, dd, *J* = 3.8, 2.2 Hz), 2.84 (4H, t, *J* = 7.7 Hz), 1.64 (6H, t, *J* = 7.1 Hz), 1.35–1.19 (16H, m), 0.83 (6H, t, *J* = 6.9 Hz); ^13^C-NMR could not be observed because of low solubility for common solvents.; HRMS (FAB): *m*/*z* 779.3196 (calcd. for C_46_H_47_N_6_O_2_S_2_ [M + H]^+^, 779.3141).

### 3.11. Synthesis of Porphycene ***1a-H_2_***

In a 100 mL three-necked round bottom flask, TiCl_4_ (0.4 mL, 3.7 mmol) was added dropwise to a THF (15 mL) solution containing Zn dust (0.48 g) and CuCl (28 mg, 0.29 mmol) at room temperature under an argon atmosphere, and then the mixture was refluxed for 2 h. Subsequently a solution of **7a** (100 mg, 0.13 mmol) in THF (15 mL) was added dropwise to the boiling reaction mixture. The mixture was stirred at the same temperature for another 1 h. After cooling to 0 °C, an aqueous 10% K_2_CO_3_ solution (18 mL) was added dropwise. After filtration the precipitate was washed with CH_2_Cl_2_. The combined organic layer was then dried with Na_2_SO_4_ and the solution was removed under reduced pressure. The crude product was purified by alumina column chromatography (CH_2_Cl_2_) and silica gel column chromatography (CH_2_Cl_2_−hexanes = 4:1). Recrystallization from CHCl_3_/methanol provided **1a-H_2_** as a green solid. Yield: 13% (13 mg, 0.02 mmol). λ_abs_ [nm] (*ε*/M^–1^·cm^–1^): 349 (4.32 × 10^4^), 407 (8.48 × 10^4^), 597 (1.03 × 10^4^), 640 (2.63 × 10^4^), 733 (7.57 × 10^3^), 805 (1.24 × 10^4^); λ_fl_ [nm] (Φ_fl_): 935 (0.16%); ^1^H-NMR (CDCl_3_, 400 MHz): δ 9.71 (2H, d, *J* = 4.0 Hz), 9.18 (1H, s), 9.03 (1H, s), 8.77 (2H, d, *J* = 4.4 Hz), 8.67 (2H, d, *J* = 4.0 Hz), 8.47 (2H, s), 8.41 (2H, s), 8.36 (2H, d, *J* = 4.4 Hz), 7.99 (2H, d, *J* = 3.3 Hz), 7.11 (2H, d, *J* = 3.3 Hz), 3.15 (4H, t, *J* = 7.5 Hz), 2.01 (4H, t, *J* = 7.7 Hz), 1.64 (4H, m), 1.50 (8H, m), 1.01 (6H, t, *J* = 7.0 Hz); ^13^C-NMR (CDCl_3_, 100 MHz) [typical signals because some signals were overlapped]: δ = 149.39, 148.09, 147.84, 145.42, 136.95, 135.45, 134.69, 133.46, 131.87, 131.15, 127.12, 126.68, 126.59, 126.11, 124.41, 123.57, 116.45, 31.93, 31.84, 30.57, 29.11, 22.76, 14.27; HRMS (FAB): *m*/*z* 745.3147 (calcd. for C_46_H_44_N_6_S_2_ [M]^+^, 745.3169).

### 3.12. Synthesis of Porphycene ***1a-Ni***

A mixture of **1a-H_2_** (3.0 mg, 4.0 μmol), Ni(OAc)_2_·4H_2_O (13 mg, 54 μmol) and pyridine (0.6 mL) in *o*-dichlorobenzene (2 mL) was heated at 180 °C for 5 h under an argon atmosphere. After cooling to room temperature, the solvent was removed under a reduced pressure. The crude product was purified by silica gel column chromatography (CH_2_Cl_2_/hexanes = 1/1) to give **1a-Ni** as a green solid. Yield: 70% yield (1.5 mg, 1.9 μmol). λ_abs_ [nm] (ε/M^–1^ cm^–1^): 348 (3.17 × 10^4^), 422 (6.90 × 10^4^), 637 (1.13 × 10^4^), 755 (8.29 × 10^3^), 837 (1.52 × 10^4^); ^1^H-NMR (CDCl_3_, 400 MHz): δ 9.93 (2H, d, *J* = 4.6 Hz), 8.52 (2H, d, *J* = 4.6 Hz), 8.42 (2H, s), 8.35 (2H, d, *J* = 5.0 Hz), 8.19 (4H, d, *J* = 3.7 Hz), 7.94 (2H, d, *J* = 3.7 Hz), 7.09 (2H, d, *J* = 3.7 Hz), 3.11 (4H, t, *J* = 7.3 Hz), 2.04–1.96 (4H, m), 1.50–1.46 (8H, m), 1.00 (6H, t, *J* = 6.9 Hz); ^13^C-NMR could not be assigned correctly because of low solubility. HRMS (MALDI-TOF): *m*/*z* 801.2339 (calcd. for C_46_H_43_N_6_NiS_2_ [M + H]^+^, 801.2331).

### 3.13. Synthesis of 2,3-Bis(5-bromo-1H-pyrrol-2-yl)-5,8-bis(3,5-dimethoxyphenyl)quinoxaline *(**3b**)*

NBS (307 mg, 1.72 mmol) was added to a solution of **2b** (550 mg, 1.03 mmol) in CHCl_3_ (55 mL) at room temperature under an argon atmosphere. The solution was stirred for 5 h under the dark and then water was added. The separated organic layer was washed with brine and dried over Na_2_SO_4_. After removal of the solvent, the crude product was purified by silica gel column chromatography (CH_2_Cl_2_). Recrystallization from CH_2_Cl_2_/methanol gave **3b** as a yellow solid. Yield: 86% (615 mg, 0.890 mmol). ^1^H-NMR (CDCl_3_, 400 MHz): δ = 9.46 (2H, s), 7.76 (2H, s), 7.10 (2H, dd, *J* = 3.8, 2.7 Hz), 6.94 (4H, *J* = 2.4 Hz), 6.61 (2H, t, *J* = 2.3 Hz), 6.20 (2H, dd, *J* = 3.8, 2.7 Hz), 3.90 (12H, s); ^13^C-NMR (CDCl_3_, 100 MHz): δ = 160.53, 140.24, 139.76, 138.46, 130.60, 129.04, 114.10, 112.19, 108.83, 103.34, 100.01, 55.73; HRMS (EI): *m*/*z* 688.0321 (calcd. for C_32_H_26_Br_2_N_4_O_2_ [M]^+^, 688.0321).

### 3.14. Synthesis of Di-tert-butyl 5,5′-(5,8-bis(3,5-dimethoxyphenyl)quinoxaline-2,3-diyl) bis(2-bromo-1H-pyrrole-1-carboxylate) *(**4b**)*

A mixture of **3b** (735 mg, 1.1 mmol), DMAP (13 mg, 0.1 mmol) and Boc_2_O (1.4 mL, 6.0 mmol) in CH_2_Cl_2_ (80 mL) was stirred for 11 h at room temperature under argon atmosphere. After removal of the solvent, the crude product was purified by recrystallization from methanol to give **4b** as a yellow solid. Yield: 89% yield (842 mg, 0.95 mmol). ^1^H-NMR (CDCl_3_, 400 MHz): δ 7.86 (2H, s), 6.98 (4H, d, *J* = 2.3 Hz), 6.55 (2H, d, *J* = 2.1 Hz), 6.27 (4H, q, *J* = 3.7 Hz), 3.81 (12H, s), 1.16 (18H, s); ^13^C-NMR (CDCl_3_, 100 MHz): δ = 160.22, 147.64, 144.53, 140.09, 139.00, 138.08, 133.57, 130.15, 116.22, 115.17, 108.98, 104.04, 100.51, 85.34, 55.37, 27.27; HRMS (ESI): *m*/*z* 911.1267 (calcd. for C_42_H_43_Br_2_N_4_NaO_8_ [M + Na]^+^, 911.1262).

### 3.15. Synthesis of Di-tert-butyl 5,5′-(5,8-bis(3,5-dimethoxyphenyl)quinoxaline-2,3-diyl)-bis(2-bromo-1H-pyrrole-1-carboxylate) *(**5b**)*

A degassed solution of **4b** (500 mg, 0.561 mmol), (1-(*tert*-butoxycarbonyl)-1*H*-pyrrol-2-yl)boronic acid (621 mg, 2.94 mmol), Pd(OAc)_2_ (5.0 mg, 0.0223 mmol), SPhos (16.0 mg, 0.0390 mmol), K_3_PO_4_ (467 mg, 2.20 mmol) in 1-butanol (10 mL) and H_2_O (3.6 mL) was heated at 80 °C for 24 h under an argon atmosphere. After cooling to room temperature, the reaction mixture was diluted with CHCl_3_. The organic layer was washed with water and brine, and dried over Na_2_SO_4_. After removal of the solvent under a reduced pressure, the crude product was purified by silica gel column chromatography (CH_2_Cl_2_). Recrystallization from methanol gave **5b** as an orange solid. Yield: 97% (576 mg, 0.542 mmol). ^1^H-NMR (CDCl_3_, 400 MHz): δ 7.77 (2H, s), 7.39 (2H, m), 6.90 (4H, d, *J* = 2.3 Hz), 6.48 (2H, t, *J* = 2.3 Hz), 6.30 (2H, m), 6.21 (4H, m), 6.13 (2H, m), 3.79 (12H, s), 1.30 (18H, s), 1.00 (18H, s); HRMS (ESI): *m*/*z* 1063.4817 (calcd. for C_60_H_67_N_6_O_12_ [M + H]^+^, 1063.4811).

### 3.16. Synthesis of 2,3-Di(1H,1′H-[2,2′-bipyrrol]-5-yl)-5,8-bis(3,5-dimethoxyphenyl)quinoxaline *(**6b**)*

A suspended solution of **5b** (560 mg, 0.58 mmol) in ethylene glycol (60 mL) was degassed for 1 h then purged with an argon atmosphere. The resulting mixture was heated at 180 °C for 2 h. After cooling to room temperature, the reaction mixture was extracted with CHCl_3_. The organic layer was washed with water and brine, and dried over Na_2_SO_4_. After removal of the solvent under a reduced pressure, the crude product was recrystallized from CH_2_Cl_2_/methanol to give **6b** as a red solid. Yield: 89% (310 mg, 0.47 mmol). ^1^H-NMR (DMSO-*d*_6_, 400 MHz): δ 9.74 (2H, s), 8.56 (2H, s), 7.73 (2H, s), 7.31 (2H, dd, *J* = 3.7, 2.3 Hz), 7.05 (2H, d, *J* = 2.3 Hz), 6.85 (2H, m), 6.65 (2H, t, *J* = 2.3 Hz), 6.40–6.42 (2H, m), 6.32–6.28 (4H, m), 3.92 (12H, s); ^13^C-NMR (DMSO-*d*_6_, 100 MHz): δ 160.02, 142.91, 140.02, 137.78, 136.99, 129.35, 128.83, 127.93, 124.58, 118.53, 113.01, 108.82, 108.70, 104.80, 103.96, 99.52, 79.17, 55.25, 22.07; HRMS (ESI): *m*/*z* 663.2720 (calcd. for C_40_H_35_N_6_O_4_ [M + H]^+^, 663.2714).

### 3.17. Synthesis of 5′,5‴-(5,8-Bis(5-hexylthiophen-2-yl)quinoxaline-2,3-diyl) bis((1H,1′H-[2,2′-bipyrrole]-5-carbaldehyde)) *(**7b**)*

POCl_3_ (0.2 mL, 2.1 mmol) was slowly added to DMF (1.4 mL, 17.9 mmol) at 0 °C under an argon atmosphere to prepare the Vilsmeier’s reagent. A solution of **6b** (60 mg, 0.090 mmol) in DMF (4.0 mL) was carefully added to the Vilsmeier’s reagent at 0 °C. The resulting mixture was stirred for 1 h at 0 °C. The concentrated aqueous acetyl acetate was added and stirred for 4 h at room temperature. The reaction mixture was extracted with CHCl_3_ and the combined organic layer was washed with water and brine, then dried over Na_2_SO_4_. After removal of the solvent under a reduced pressure, the crude product was recrystallized from CH_2_Cl_2_/methanol to give **7b** as a red sold. Yield: 57% (37 mg, 0.051 mmol). ^1^H-NMR (DMSO-*d*_6_, 400 MHz): δ 12.37 (2H, s), 11.41 (2H, s), 9.40 (2H, s), 7.92 (2H, s), 7.08–7.05 (6H, m), 6.86–6.84 (2H, m), 6.64–6.62 (4H, m), 6.38–6.36 (2H, m), 3.80 (12H, s); ^13^C-NMR could not be observed because of low solubility for common solvents.; HRMS (ESI): *m*/*z* 719.2618 (calcd. for C_42_H_35_N_6_O_6_ [M + H]^+^, 719.2613).

### 3.18. Synthesis of Porphycene ***1b-H_2_***

In a 100 mL three-necked round bottom flask, TiCl_4_ (0.2 mL, 2.2 mmol) was added dropwise to a THF (8 mL) solution containing Zn dust (0.30 g) and CuCl (17 mg, 0.29 mmol) at room temperature under argon atmosphere, then the mixture was refluxed for 2 h. Subsequently a solution of **7b** (55 mg, 0.077 mmol) in THF (8 mL) was added dropwise to the boiling reaction mixture. The mixture was stirred at the same temperature for another 1 h. After cooling to 0 °C, an aqueous 10% K_2_CO_3_ solution (10 mL) was added dropwise. After filtration the precipitate was washed with CH_2_Cl_2_, the combined organic layer was then dried with Na_2_SO_4_, and the solution was removed under reduced pressure. The crude product was purified by alumina column chromatography (CH_2_Cl_2_) and silica gel column chromatography (CH_2_Cl_2_−hexanes = 1:4). Recrystallization from CHCl_3_/methanol provided **1b-H_2_** as a green solid. Yield: 17% (9 mg, 0.013 mmol). λ_abs_ [nm] (ε/M^−1^ cm^–1^): 344 (3.86 × 10^4^), 397 (1.46 × 10^5^), 585 (1.23 × 10^4^), 632 (3.80 × 10^4^), 710 (1.14 × 10^4^), 781 (2.11 × 10^4^); λ_fl_ [nm] (Φ_fl_): 911, 980 (0.20%); ^1^H-NMR (CDCl_3_, 400 MHz): δ 9.52 (2H, d, *J* = 5.0 Hz), 8.91 (2H, d, *J* = 4.1 Hz), 8.82 (2H, d, *J* = 4.1 Hz), 8.62 (2H, s), 8.52 (2H, d, *J* = 4.6 Hz), 8.38 (2H, s), 7.45 (4H, d, *J* = 2.3 Hz), 6.82 (4H, t, *J* = 2.5 Hz), 4.04 (12H, s); ^13^C-NMR could not be assigned correctly because of low solubility.

### 3.19. Synthesis of Porphycene ***1b-Ni***

A mixture of **1b-H_2_** (1.0 mg, 1.5 μmol), Ni(OAc)_2_·4H_2_O (7.5 mg, 30 μmol) and pyridine (0.3 mL) in o-dichlorobenzene (1 mL) was heated at 180 °C for 6 h under an argon atmosphere. After cooling to room temperature, the solvent was removed under a reduced pressure. The crude product was purified by silica gel column chromatography (CH_2_Cl_2_/hexanes = 1/1) to give **1b-Ni** as a green solid. Yield: 45% yield (0.5 mg, 0.674 μmol). *λ*_abs_ [nm] (*ε*/M^−1^ cm^−1^): 347 (1.07 × 10^4^), 412 (2.88 × 10^4^), 622 (4.86 × 10^3^), 735 (4.29 × 10^3^), 812 (8.53 × 10^3^); ^1^H-NMR (400 MHz, CDCl_3_): δ 9.53 (2H, d, *J* = 5.0 Hz), 9.41 (2H, s), 9.33 (2H, s), 8.92 (2H, d, *J* = 3.2 Hz), 8.83 (2H, d, *J* = 4.1 Hz), 8.62 (2H, s), 8.52 (2H, d, *J* = 2.3 Hz), 8.38 (2H, s), 7.45 (4H, d, *J* = 2.3 Hz), 6.83 (2H, t, *J* = 2.5 Hz), 4.04 (12H, s): ^13^C-NMR could not be assigned correctly because of low solubility.

## 4. Conclusions

In summary, the quinoxaline-fused porphycenes **1a-H_2_** and **1b-H_2_** and their nickel complexes were successfully synthesized and characterized. These porphycenes displayed absorption and fluorescence at the NIR regions by the introduction of quinoxaline parts onto the porphycenes because of the effective expansion of the π-conjugation. The attached quinoxaline moiety also contributes to a decrease in the reduction potentials. We revealed that protonation by addition of TFA conducted for the dicationic porphycene from free base **1a-H_2_** and **1b-H_2_** and dicationic quinoxaline from nickel complexes associated with generation of the NIR absorptions. These results demonstrated that the synthesis of porphycene–based NIR absorbing dyes offers the practical applications for PDT, non-linear optical and OSC materials.

## Figures and Tables

**Table:** 

Compound	1	2	3
**Pc**	–15.62	–	–
**1a-H_2_**	–8.51	–12.56	–8.57
**1b-H_2_**	–8.57	–12.67	–9.03

## Supplementary Materials

The following are available online: Table S1 and S2, and Figure S1: Crystal data of **2a** and **2b**; Figure S2 and S3: H-H COSY and NOE spectra of **1a-H_2_**; Figure S4 and S5: Changing of the absorption spectra of THPc and THPc-Ni upon addition of TFA.
