# Supplementary material for: Synthesis, Characterization and Protonation Behavior of Quinoxaline-Fused Porphycenes
Source: Molecules. 2017 May 31;22(6):908. doi: 10.3390/molecules22060908 (PMC6152776; doi:10.3390/molecules22060908)
Supplement: Supplementary file 1 [file molecules-22-00908-s001.pdf]

Supporting Information

Synthesis, Characterization and Protonation behaviors of Quinoxaline-Fused Porphycenes

Daiki Kuzuhara*, Mika Sakaguchi, Wataru Furukawa, Takuya Okabe, Naoki Aratani, and Hiroko Yamada*

Graduate School of Materials Science, Nara Institute of Science and Technology, 8916-5 Takayama-cho, Ikoma, Japan; hyamada@ms.naist.jp

Supporting information

Table 1. Crystal data and structure refinement for **2a**.

Empirical formula	C36 H40 N4 S2	
Formula weight	592.84	
Temperature	90 K	
Wavelength	0.71073 Å	
Crystal system	Monoclinic	
Space group	*C*2/*c*	
Unit cell dimensions	*a* = 37.389(4) Å	
	b = 5.1476(5) Å	β = 93.283(2)°
	c = 32.260(3) Å	
Volume	6198.7(11) $Å^3$	
Z	8	
Density (calculated)	1.270 g/cm^3	
Absorption coefficient	0.204 mm^{-1}	
F(000)	2528	
Crystal size	0.10 x 0.05 x 0.05 mm^3	
Theta range for data collection	1.62 to 26.92°	
Index ranges	$-47 \leq h \leq 45$, $-4 \leq k \leq 6$, $-31 \leq l \leq 41$	
Reflections collected	18346	
Independent reflections	6704 [*R*(int) = 0.0495]	
Completeness to theta = 26.92°	99.7%	
Absorption correction	Empirical	
Max. and min. transmission	0.9899 and 0.9799	
Refinement method	Full-matrix least-squares on F^2	
Data / restraints / parameters	6704 / 0 / 410	
Goodness-of-fit on F^2	1.009	
Final R indices [$I > 2\sigma(I)$]	R_1 = 0.0428, wR_2 = 0.0919	
R indices (all data)	R_1 = 0.0739, wR_2 = 0.1088	
Largest diff. peak and hole	0.439 and –0.267 e.$Å^{-3}$	
CCDC No. 1543212		

Table 2. Crystal data and structure refinement for **2b**.

Empirical formula	C32 H28 N4 O4
Formula weight	532.58
Temperature	90 K
Wavelength	0.71073 Å
Crystal system	Monoclinic
Space group	$P2_1/c$
Unit cell dimensions	a = 11.750(2) Å
	b = 10.982(2) Å β = 104.032(4)°
	c = 21.089(5) Å
Volume	2640.3(9) $Å^3$
Z	4
Density (calculated)	1.340 g/cm^3
Absorption coefficient	0.090 mm^{-1}
F(000)	1120
Crystal size	0.30 x 0.30 x 0.02 mm^3
Theta range for data collection	1.79 to 24.00°
Index ranges	$-13 \le h \le 11, -12 \le k \le 12, -14 \le l \le 24$
Reflections collected	12564
Independent reflections	4160 [R(int) = 0.0943]
Completeness to theta = 24.00°	100.0%
Absorption correction	Empirical
Max. and min. transmission	0.9982 and 0.9735
Refinement method	Full-matrix least-squares on F^2
Data / restraints / parameters	4160 / 0 / 365
Goodness-of-fit on F^2	1.095
Final R indices [$I > 2\sigma(I)$]	R_1 = 0.0727, wR_2 = 0.1849
R indices (all data)	R_1 = 0.1405, wR_2 = 0.2184
Largest diff. peak and hole	1.470 and –0.276 e.$Å^{-3}$
CCDC No. 1543211	

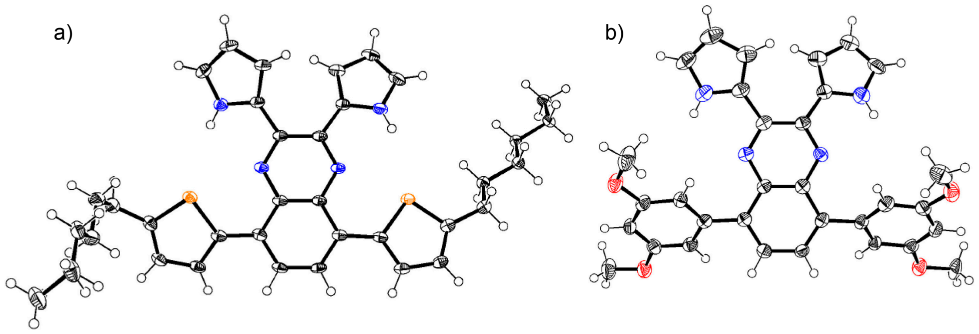

Figure S1. Crystal structures of **2a** and **2b**. Thermal ellipsoids represent for 50% probability.

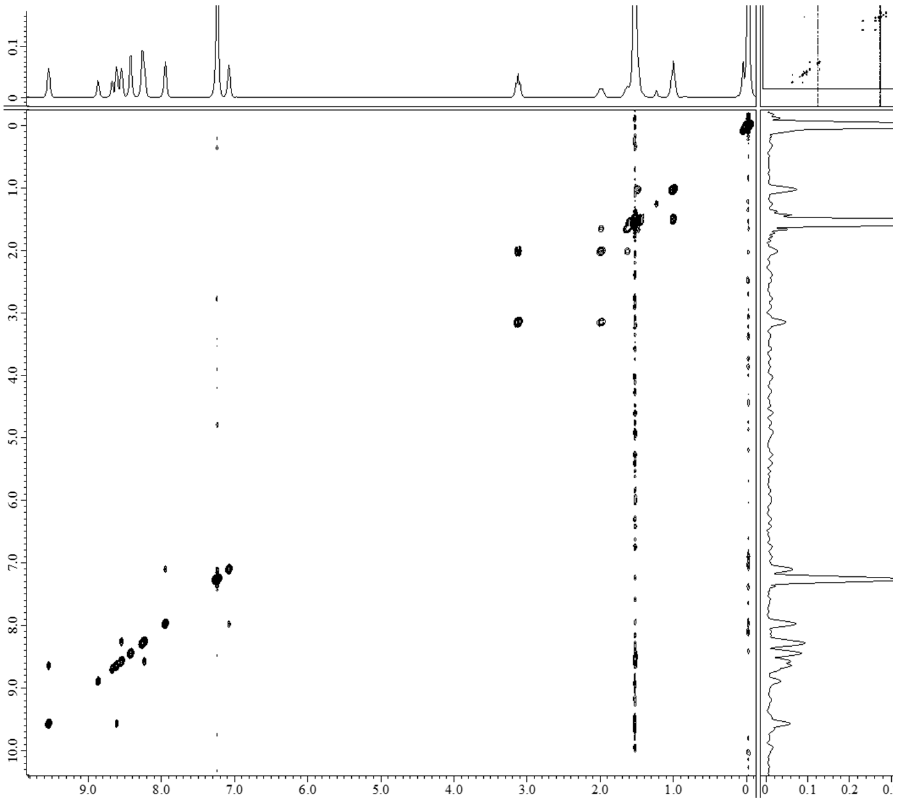

Figure S2. H-H-COSY spectrum of **1a-H$_2$** in $CDCl_3$.

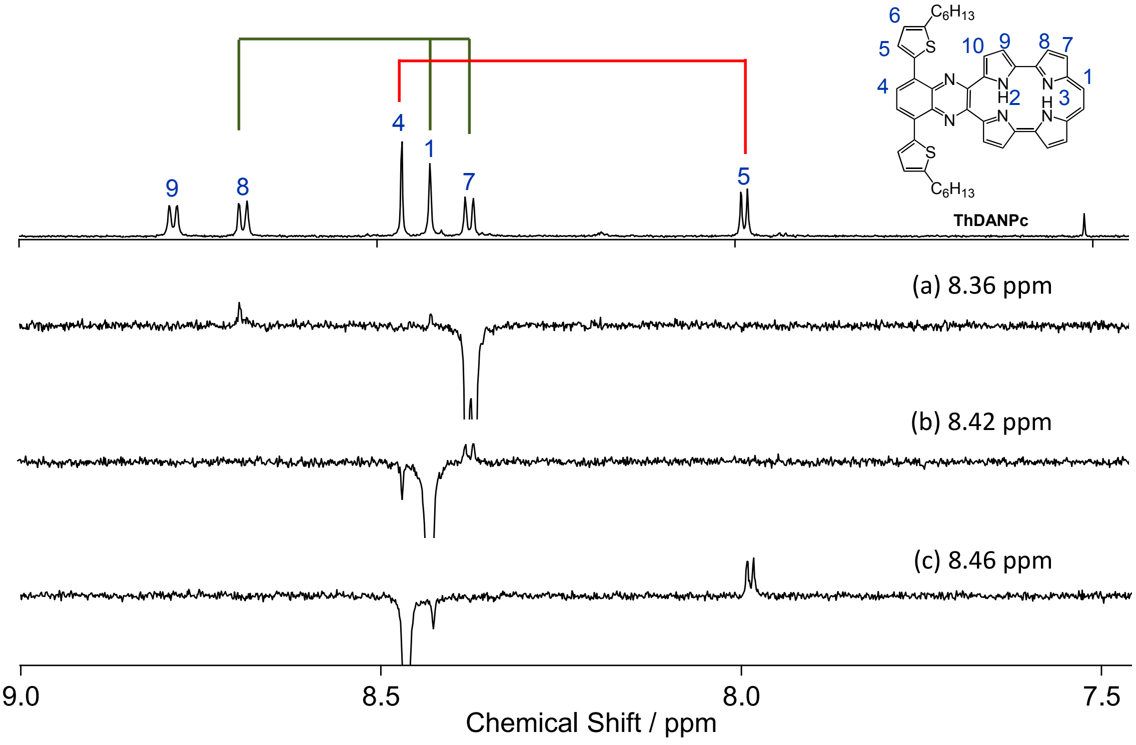

Figure S3. NOE spectra of **1a-H$_2$** in $CDCl_3$.

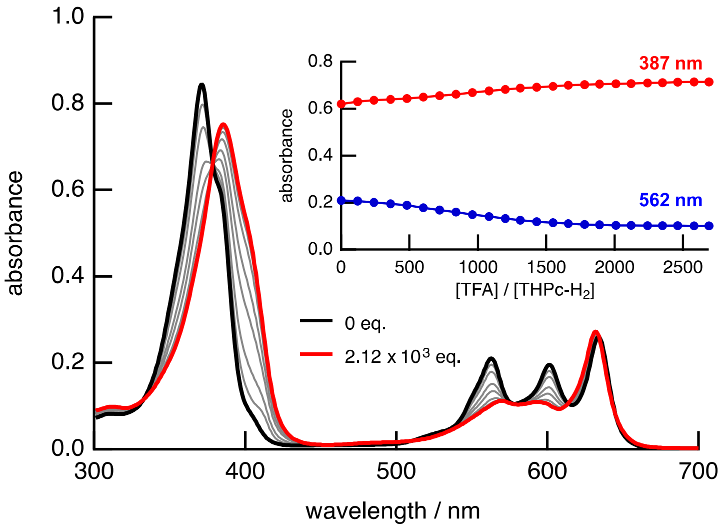

Figure S4. Changing of the absorption spectra of **THPc-H$_2$** upon addition of the TFA.

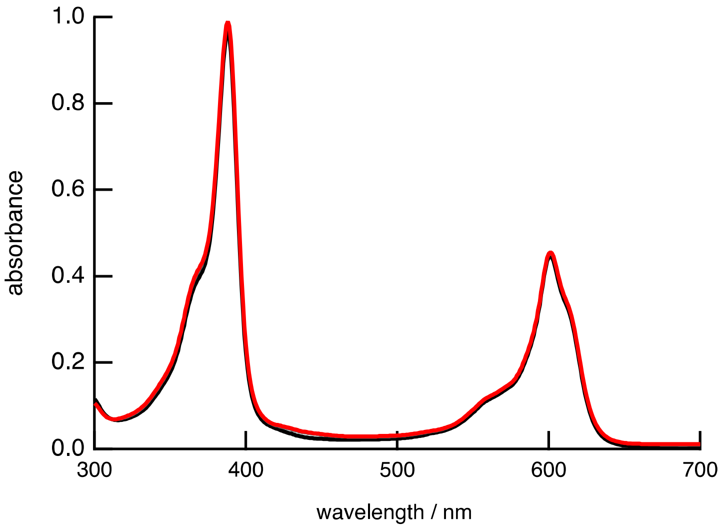

Figure S5. Changing of the absorption spectra of **THPc-Ni** upon addition of the TFA.
